# Microstructure Evolution and Toughening Mechanism of a Nb-18Si-5HfC Eutectic Alloy Created by Selective Laser Melting

**DOI:** 10.3390/ma15031190

**Published:** 2022-02-04

**Authors:** Longhui Yao, Liang Wang, Xiaojiao Song, Ran Cui, Binqiang Li, Qi Lv, Liangshun Luo, Yanqing Su, Jingjie Guo, Hengzhi Fu

**Affiliations:** 1 National Key Laboratory for Precision Hot Processing of Metals, School of Materials Science and Engineering, Harbin Institute of Technology, Harbin 150001, China; yaolh@hit.edu.cn (L.Y.); cuiran@hit.edu.cn (R.C.); rijvaewq@gmail.com (B.L.); 20B909063@hit.edu.cn (Q.L.); luols@hit.edu.cn (L.L.); suyq@hit.edu.cn (Y.S.); wanxguan@gmail.com (J.G.); roiakamas@gmail.com (H.F.); 2 Department of Materials Processing, Shanghai Space Propulsion Technology Research Institute, Shanghai 201109, China; kipcirat@gmail.com; 3 State Key Laboratory of Advanced Welding and Joining, School of Materials Science and Engineering, Harbin Institute of Technology, Harbin 150001, China

**Keywords:** selective laser melting (SLM), niobium–silicon high-temperature alloy, hafnium carbide dispersion, fracture toughness

## Abstract

Because of their superior mechanical performance at ultra-high temperatures, refractory niobium–silicon-based alloys are attractive high-temperature structural alloys, particularly as structural components in gas turbine engines. However, the development of niobium–silicon-based alloys for applications is limited because of the trade-off between room temperature fracture toughness and high-temperature strength. Here, we report on the fabrication of a Nb-18Si alloy with dispersion of hafnium carbide (HfC) particles through selective laser melting (SLM). XRD and SEM-BSE were used to examine the effects of scanning speed on the microstructure and the phase structure of the deposited Nb-18Si-5HfC alloy. The results show that when the scanning speed rises, the solid solubility of the solid solution improves, the interlamellar spacing of eutectics slowly decrease into nano-scale magnitude, and the corresponding hafnium carbide distribution becomes more uniform. We also discover the hafnium carbide particles dispersion in the inter-lamella structure, which contributes to its high fracture toughness property of 20.7 MPa∙m^1/2^ at room temperature. Hardness and fracture toughness are simultaneously improved because of the control of microstructure morphology and carbide distribution.

## 1. Introduction

Because of their high melting points (>1700 °C), appropriate densities, and excellent and balanced mechanical properties, refractory Nb-Si-based ultra-high-temperature alloys have become extremely appealing materials [1,2,3,4]. The very low tuned room-temperature fracture toughness of niobium–silicon-based ultra-high-temperature alloys of application, however, is a major issue [5,6]. The consensus among academics is that alloying can improve the toughness of Nb-Si-based ultra-high-temperature alloys [7]. The researchers discovered that the elements Ti [8], Hf [9], and Ga [5] typically improve the fracture toughness of Nb-Si-based ultra-high-temperature alloys by refining the lamellae and increasing the number of grain boundaries. However, further increases in the fracture toughness of Nb-Si-based ultra-high-temperature alloys may be hampered by a loss of strength at high temperatures or a loss of resistance to oxidation.

The rapid solidification process has an ultra-high temperature gradient and a very fast cooling rate, making it the most promising method for improving fracture toughness at room temperature without compromising high-temperature oxidation resistance [10,11]. The microstructure of Nb-Si-based ultra-high-temperature alloys can be significantly refined through rapid solidification methods, such as additive manufacturing [12,13], laser/electron beam surface melting [14], and powder metallurgy methods [15]. Among them, additive manufacturing has attracted more and more attention because it can prepare metallic products with complex constitutional designs and shapes. Zhang et al. [11], for example, used a selective laser melting technique to create a dense Nb-18Si-based ultra-high-temperature alloy. The oxidation kinetic rate of the Nb-18Si alloy formed by laser selective melting is substantially lower than that of the alloy fabricated by the vacuum arc melting (VAM) technique. In the 50 h oxidation test at 1250 °C, the weight gain of the Nb-18Si, prepared by SLM, is 96.48 mg/cm^2^, which is less than half of the weight gain of the alloy prepared the VAM. The niobium–silicon alloy, prepared by additive manufacturing, not only has excellent oxidation resistance, but is also outstanding in improving the room-temperature fracture toughness. Huang et al. [16] used laser directed energy deposition to investigate the influence of Mo on the microstructure and fracture toughness of the Nb-Si alloy. As a result, the room-temperature fracture toughness of the Nb-Si alloy was adjusted from 8 to 12.4 MPa∙m^1/2^ based on the molybdenum volume fraction added. Although researchers have some understanding of the properties of niobium–silicon alloys produced by additive manufacturing, there are relatively few reports in the literature on the preparation of niobium–silicon alloys produced by additive manufacturing. This is mainly due to the large difference in the physical and chemical properties of the various components of the Nb-Si alloy, which is not conducive to metallurgy. Because of this, we are particularly interested in understanding the mechanism by which scanning speed affects the development of the microstructure of niobium–silicon alloys and the distribution of carbides in them.

In this work, we prepared Nb-18Si-5HfC ultra-high-temperature alloys by controlling the processing parameters of SLM. The microstructure and fracture toughness characterization were systematically studied. In-depth analyses were conducted on the relationship between processing parameters, microstructure evolution, and fracture behavior. 

## 2. Experimental

### 2.1. Preparation of Powders and SLM Processing

It was decided that we would employ commercially pure elemental powders for this investigation, which included Nb powder (99.7 percent at.; percent purity, 60–80 μm), Si powder (98.9 percent at.; percent purity, 13–20 μm), and HfC powder (99.9 percent at.; percent purity, 8–13 μm), among others. The composite powder was uniformly mixed in a laboratory ball mill (a planetary ball mill PMQW4 from Nanjing Chi Shun Technology Development Co., Ltd.), and argon gas was introduced into the ball mill tank to prevent the powder from oxidizing during the ball milling process. We employed C15 carbon steel balls with a diameter of 10 mm, with a ball-to-powder weight ratio of 5:1. Following that, mixing was carried out at a constant speed of 200 rpm for 3 h to obtain uniformly distributed Nb-18Si-5HfC powder, which was then dried. Using a Harbin FORWARD LM280 equipment (Harbin Foward Multidimensional Intelligent Equipment Co., LTD, Harbin, China), equipped with a 500-watt Yb:YAG fiber laser with a 70-micron spot size, a cubic sample with dimensions of 10 × 10 × 10 mm^3^ was created under the protection of a high-purity argon environment. The substrate was a pure titanium plate with dimensions of 150 mm × 100 mm and a thickness of 15 mm. Prior to the SLM process, the surface of the substrate was polished with abrasive paper to remove the oxidation deposit and washed with acetone to prepare it for the SLM process. The Nb-18Si-5HfC (in at.%: 17.8 Si, 4.88 HfC, 0.143 O, 0.015 C, 0.003 H, 0.007 N, balance Nb) samples were created using a variety of laser scanning rates ranging from 600 to 1200 mm s^−1^, a set layer thickness of 60 μm, and a constant hatching distance of 100 μm for each layer. The layers were scanned using a continuous laser mode with a fixed power of 380 W in a zigzag pattern that was alternated at 67° between each layer, with the intensity of the laser remaining constant throughout.

### 2.2. Determination of the Phase and Examination of the Microstructure

Pure elemental powders and the print sample were subjected to X-ray diffraction (XRD) measurements on a Philips X’Pert X-ray diffractometer with Cu Kα radiation, a tube voltage of 40 kV, and a tube current of 40 mA. Scanning electron microscopy with Z-sensitive backscatter electron (BSE) contrast (JEOL JXA-8230, JEOL Ltd., Akishima, Japan) was used to examine the morphology of the molten pool, nano-indentation, and microstructures of Nb-18Si-5HfC. An electron probe micro-analysis (EPMA) JEOL JXA-8230, equipped with wavelength dispersive X-ray spectrometers, was used to analyze the elemental composition distribution data of the phase using the following settings: E = 10 keV and i = 0.48 nA. EPMA differentiates between elements using the wavelength of their characteristic X-ray emissions.

### 2.3. Mechanical Properties

In order to evaluate the fracture toughness, a room-temperature indentation test was carried out according to the established test method suitable for brittle materials and small-scale specimens [17,18,19,20]. The Vickers hardness is determined under a load of 1 kg with a diamond pyramid indenter using a microhardness testing equipment HVS-1000A, (Ningbo kecheng instrument Co., Ltd., Ningbo, China) with the loading duration being 20 s. The Nb-18Si-5HfC alloys also underwent nano-indentation tests to determine their microstructure, nano-hardness, and modulus of elasticity. The nano-hardness testing was carried out using an Agilent Nano Indenter G200 system (Agilent, Santa Clara, CA, USA). The Berkovich diamond indenter was used to carry out the nano-indentation process. Individual area indentation was carried out in displacement-controlled mode up to a 500 nm range, with the loading and unloading rates maintained at a value of 10 nm/s and the loading duration set at 5 s.

## 3. Results

**Figure 1:**
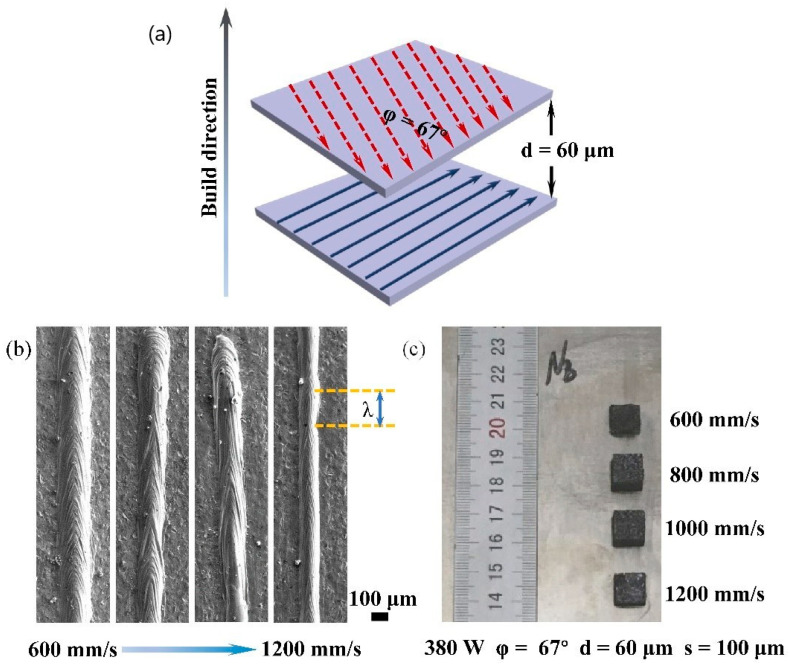
(**a**) Schematic illustration of meandering scanning strategy; (**b**) single-pass melt pool morphology at different scanning speeds; (**c**) schematic illustration of the as-printed 1 cm^3^ cubic Nb-18Si-5HfC alloys cubic specimen on the titanium plate.

Figure 1 shows a schematic diagram of the building layout and printed object. Following each layer, a rotation angle of 67° was applied following a meandering laser scanning strategy, as shown in the illustration in Figure 1a. A single-pass molten pool is displayed in Figure 1b with varying scan speeds. The melt track width reduces from 200 ± 5 μm to 100 ± 3.8 μm when the scan speed rises from 600 mm/s to 1200 mm/s. At a speed of 600 mm/s, the fish scale pattern’s wavelength is determined to be steady. The wavelength of the scale pattern reveals more and more aperiodic instability as the scanning speed rises. These observations indicate that these fluctuations may be caused by the instability of melt flow in the molten pool and will further affect the quality of multi-track molding. Figure 1c shows the top surface morphology of the block at various scanning rates. When the fish scale wavelength is steady, the surface of the block is smooth. This shows that adjusting the scan speed can improve the forming quality of Nb-18Si-5HfC.

**Figure 2:**
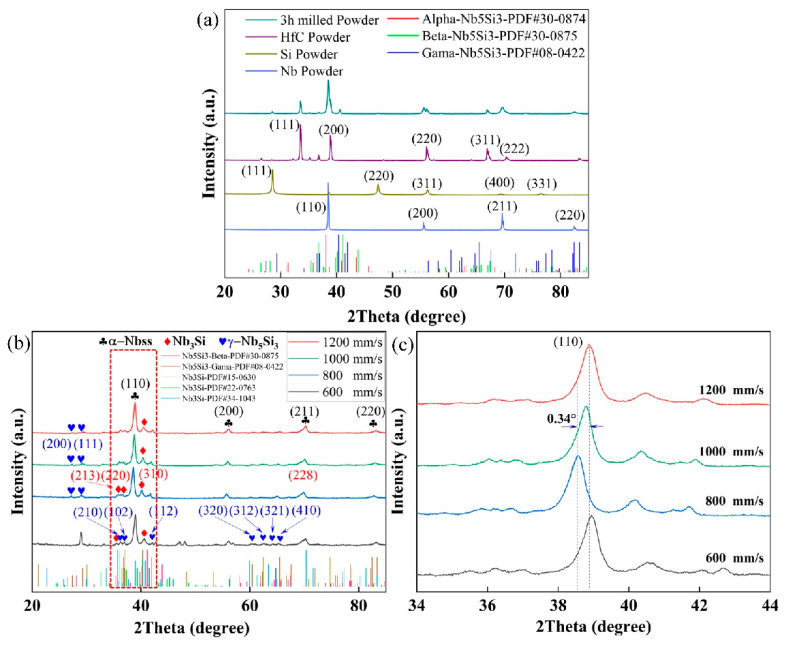
X-ray diffraction patterns of (**a**) powders after 3 h of ball milling; (**b**) component phases present in as-deposited Nb-18Si-5HfC alloys at various scan speeds; (**c**) local magnification of the diffraction peak shift at various scan speeds.

The XRD patterns of elemental powders and samples deposited by SLM are shown in Figure 2. Figure 2a gives the standard PDF (PDF#30-0874, PDF#30-0875, PDF#08-0422) card of the niobium intermetallic compounds that may be formed during the ball milling of the composite powder. Typical 3-principal intensity diffraction peaks of the intermetallic compound were not observed in the powder after 3 h of ball milling, as shown in Figure 2a. This indicates that no metallurgical reaction occurred in the ball mill powder to form the intermetallic compounds. According to the XRD patterns presented in Figure 2b, the Nb-Si-based alloy, composed of Nbss, Nb_5_Si_3_, and Nb_3_Si, was effectively produced through SLM. Further investigation of the diffraction peak location reveals that the scanning speed has a considerable influence on the interplanar spacing, as seen in Figure 2c. When the scanning speed exceeds 600 mm/s, the diffraction peak moves to the lower 2θ values. The shift of diffraction peaks is mainly caused by solid solution, internal stresses, and lattice defects [21]. The surface of the sample, prepared by selective laser melting, is usually subjected to tensile stress [22]. Therefore, internal stress and lattice defects in the above cases will increase the spacing of Nb crystal planes. When the scanning speed was raised progressively from 800 mm/s to 1200 mm/s, the diffraction peak migrated toward higher 2θ values, 0.34°. The primary explanation for the rightward shift of the diffraction peaks with increased scanning speed can be traced to the shrinkage of the Nb lattice caused by the dissolution of Si atoms with small atomic radius into the Nb bcc lattice. This discovery implies that, as the scanning speed rises, the supersaturation of the α-Nb by Si solutes increases.

**Figure 3:**
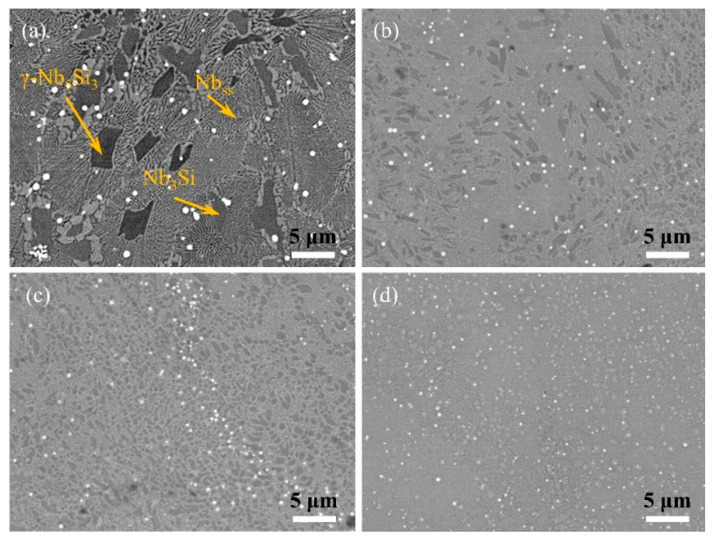
BSE pictures of the microstructure of Nb-18Si-5HfC-based alloys deposited at various scanning speeds: (**a**) 600 mm/s, (**b**) 800 mm/s, (**c**) 1000 mm/s, (**d**) 1200 mm/s.

**Figure 4:**
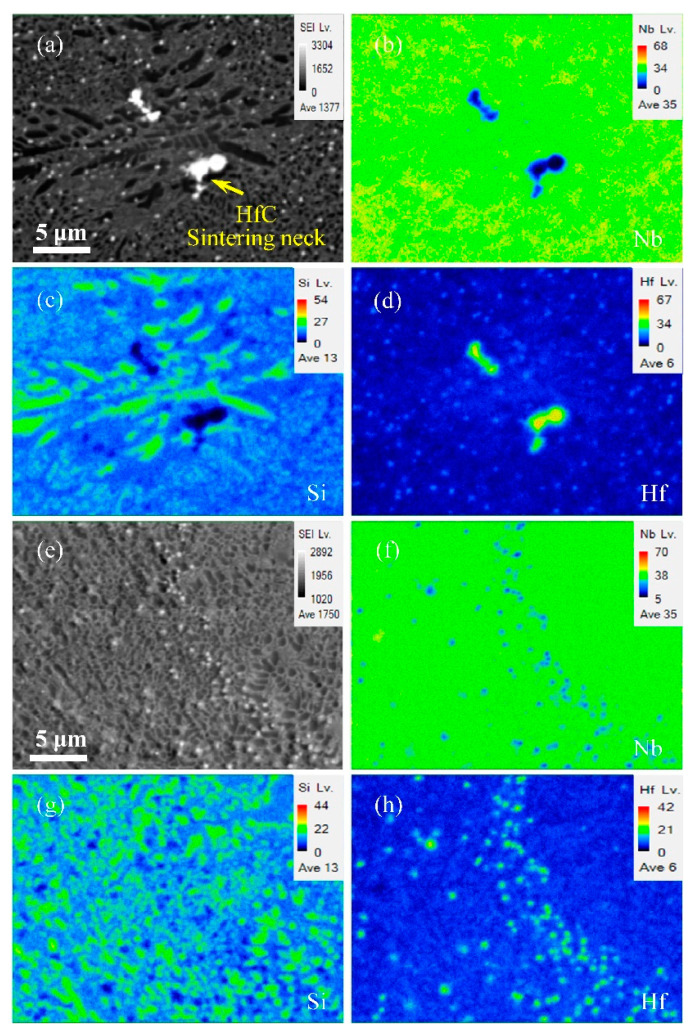
Results of the EPMA experiments. (**a**) BSE image showing the location of the EPMA maps at scanning speed 600 mm/s; (**b**–**d**) the corresponding elemental concentration distribution mappings of Nb, Si and Hf; (**e**) BSE image showing the location of the EPMA maps at scanning speed 1000 mm/s; (**f**–**h**) the corresponding elemental concentration distribution mappings of Nb, Si, and Hf.

Figure 3 illustrates the BSE picture of the alloy after SLM processing at various scanning speeds. As scanning speed increased, a significant difference in microstructure occurred. When the adopted speed was 600 mm/s, the contrast of the microstructure was obvious. The phase constituent in the microstructures includes the grey Nbss, dark grey Nb_3_Si, and black λ-Nb_5_Si_3_, as seen in Figure 3a. When the scanning speed was increased to 800 mm/s, seen in Figure 3b, the microstructure was composed of Nbss and Nb_3_Si. As the scanning speed was increased up to 1000 mm/s, the aspect ratio of Nb_3_Si was about 1.5 and its the distribution was more uniform, as shown in Figure 3c. When the scanning speed was at 1200 mm/s, the homogeneous microstructure was achieved and the nano-lamellar Nbss/Nb_3_Si eutectics was formed, as shown in Figure 3d. Interestingly, as the scanning speed increased, the particle size of HfC reduced, and its distribution became more uniform, which indicated successful preparation of the HfC uniformly distributed nano-lamellar eutectic Nb-18Si-5HfC alloy by using SLM. In addition, in order to characterize the effect of scanning speed on the microstructure morphology and element distribution, EPMA analysis was performed, as shown in Figure 4. When the scanning speed was 600 mm/s, the hafnium carbide particles were sintered, and the silicide was relatively coarse. When the scanning speed was 1000 mm/s, the hafnium carbide particles were finely dispersed.

**Figure 5:**
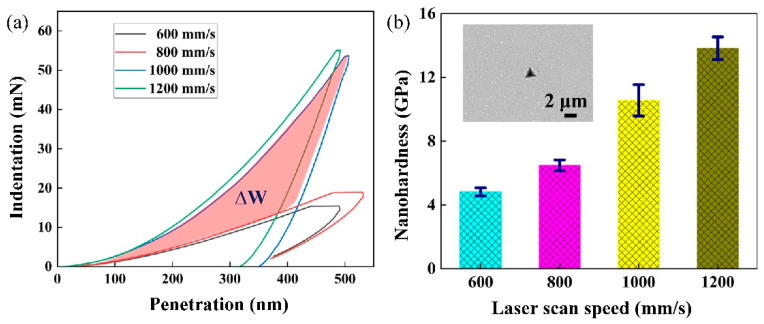
(**a**) Representative nano-indentation load–depth curves for SLM-processed Nb-18Si-5HfC alloys; (**b**) average nano-hardness with different scan speeds (the inset shows a typical nano-indentation morphology).

The hardness and elastic modulus of the Nb-18Si-5HfC were measured by using the nano-indentation test. Figure 5a depicts the nano-indentation load–displacement curve measured on a smooth-surface Nb-18Si-5HfC sample deposited by selective laser melting. As depicted in the inset of Figure 5b, the total length of the indentation and its surrounding strain field is approximately 2 μm. Indentation load increased significantly with the scanning speed, varying from 15.5 mN at 600 mm/s to 54.9 mN at 1200 mm/s, as shown in Figure 5a. The corresponding dynamic nano-hardness Hv values (4.81 GPa at 600 mm/s and 13.87 GPa at 1200 mm/s), as revealed in Figure 5b. The increase in nano-hardness is mainly attributed to the significant grain refinement effect in the selective laser melting process. As the scanning speed improved, the absorption function, ∆W, increased significantly, as shown in the red area in Figure 5a. This implies that the fracture toughness also improved at the same time.

**Figure 6:**
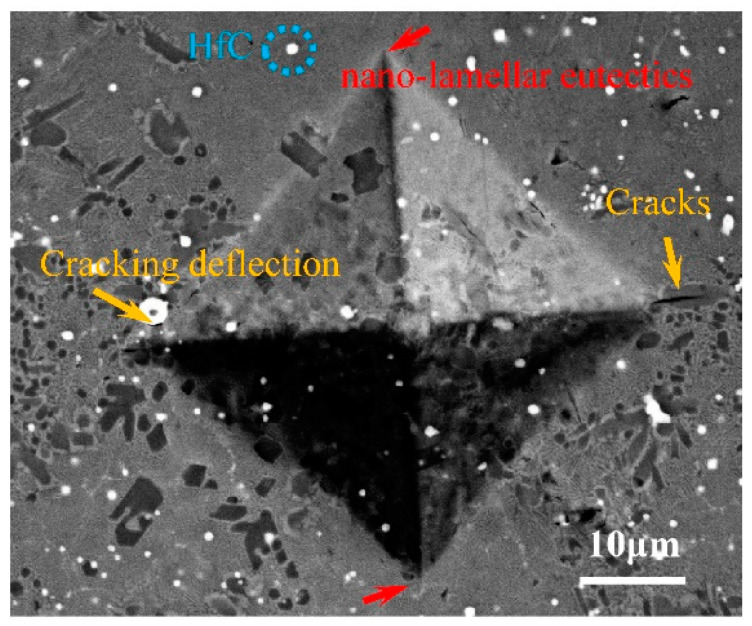
BSE-SEM micrographs of Nb-18Si-5HfC alloy scanning speed for 1000 mm/s showing microcrack deflection due to the impartation of extrinsic toughening by an HfC particle.

Figure 6 illustrates the micro-indentation method for evaluating the fracture toughness of the Nbss/Nb_3_Si eutectics created by SLM, based on nano-indentation measurements. It is important to note that the nano-indentation and micro-indentation measurement process ensure that the eutectic region is covered, thereby eliminating the effect of primary Nb_3_Si and λ-Nb_5_Si_3_ particles on fracture toughness. It is necessary to quantify the crack length and indentation dimension in order to calculate fracture toughness (*K_C_*), which is calculated using the following equation provided by Kashyap [5]:

(1) $$K_{C}=\lambda_{\nu }\cdot {\left(\frac{a}{l}\right)}^{1/2}{\left(\frac{E}{H}\right)}^{2/3}\frac{P}{C^{3/2}}$$

where *H* denotes the microhardness, *E* denotes Young’s modulus, *λ_ν_* denotes the modifying factor with value 0.015, *P* denotes the load, and *l* denotes the length of the fracture starting at the corners of Vickers indentation. The value of fracture toughness for different scanning speeds is listed in Table 1. In Figure 6, typical Vickers indentation patterns of the sample attained at a scanning speed of 1000 mm/s showed that the cracks were mainly initiated in the eutectic phase. On the contrary, there were no cracks observed in the eutectic nano-lamellar, which is denoted by a red arrow in Figure 6. At a scanning speed of 1000 mm/s, 20.7 MPa∙m^1/2^ was calculated as the indentation fracture toughness maximum.

## 4. Discussion

Two fundamental advances have been made in Nb-18Si-5HfC-based alloys. First, through the use of additive manufacturing, we created a multi-phase non-equilibrium regime (α-Nb, Nb_3_Si, Nb_5_Si_3_), not achieved by conventional synthesis and processing routes. Second, we demonstrate the possibility of improving fracture toughness by dispersing carbides through SLM.

### 4.1. Microstructure Evolution of Nb-18Si-5HfC Alloys during SLM

During SLM, there are several typical characteristics, which may influence the final microstructure and mechanical properties of the deposited samples, for example the large thermal gradient (G), rapid solidification velocity (R), high cooling rate (*v*), and complex thermal cycles, resulting in partially remelting of the deposited materials. The normal solidification velocities, Vn, of the solidification front is mathematically related to the scanning speed, Vb, through the angle, θ, with Vn = Vb·cosθ. The scanning speed has an effect on the phase transformation by varying the degree of undercooling and solidification rate. The solidification microstructure evolution in the SLM-produced Nb-18Si-5HfC alloy is consistent with the solidification microstructure selection diagram for the interfacial response function of the temperature gradient calculated from the literature [23]. The temperature at the solid/liquid interface of Nbss/Nb_3_Si eutectics for under the scanning speed ranging from 800 to 1200 mm/s is higher than that of Nbss dendrites, Nb_3_Si dendrites, λ-Nb_5_Si_3_ dendrites, and Nbss/λ-Nb_5_Si_3_ eutectics. Therefore, Nbss/Nb_3_Si eutectics precipitates preferentially during solidification at printing speed 800–1200 mm/s. The following equation describes the effect of scanning speed on thermal and kinetic undercooling in a melt pool [24]:

(2) $$\Delta T_{t}=\frac{\Delta H_{f}}{c_{p}^{l}}F_{lv}\left(P_{t}\right)$$

(3) $$P_{t}=\frac{v_{b}R}{2D_{T}}$$

(4) $$\Delta T_{k}=\frac{v_{b}}{\lambda }$$

(5) $$\lambda =\frac{\Delta H_{f}v_{0}}{k_{B}T_{L}^{2}}$$

where $\Delta H_{f}$ denotes the heat of fusion (J mol^−1^), $c_{p}^{l}$ denotes the specific heat of the liquid (J mol^−1^ K^−1^), $F_{lv}\left(P_{t}\right)$ denotes the Ivantsov function, $P_{t}$ denotes the thermal Pe’clet number, R denotes the curvature radius of the crystal tip (m), $D_{T}$ denotes the thermal diffusivity (m^2^ s^−1^), λ denotes the interfacial kinetic coefficient, $v_{0}$ denotes the speed of sound (m s^−1^), $k_{B}$ denotes the Boltzmann constant, and $T_{L}$ denotes the liquidus temperature (K).

When the applied laser scanning speed was increased, the solidification rate of the liquid front inside the melt pool ascended, leading to an acceleration of both thermal undercooling (Equations (1) and (2)) and kinetic undercooling (Equations (1) and (2)). As a result, as the scanning speed rose from 800 mm/s to 1200 mm/s, the Nbss/Nb_3_Si eutectic content progressively increased, while the eutectic lamellar spacing gradually reduced (Figure 3). The significant increase in solute capture occurred as a consequence of the greatly increased temperature gradients inside the melt pool, which resulted in a rightward shift of the diffraction peak of 2θ at a higher scan speed (Figure 2).

Moreover, when the scanning speed was 600 mm/s, the effect of thermal cycling on the microstructure was not negligible. On the one hand, the presence of thermal cycling caused the microstructure to undergo in situ heat treatment effects, generating λ-Nb_5_Si_3_ dendrites. On the other hand, the size of HfC particles increased significantly. Coalescence or Ostwald ripening may coarsen carbides [25]. Coalescence mobile, caused by thermal cycling, disperse HfC contacts with each other and coalesce by a sintering mechanism to form one new HfC, as shown in Figure 4a–d. Under such conditions, the microstructure and carbide distribution are not conducive to improving fracture toughness. Furthermore, when the scanning speed was increased to 1000 mm/s, the hafnium carbide particles were distributed more uniformly and diffusely, and the fracture toughness increased significantly. 

### 4.2. Toughening Mechanism of Nb-18Si-5HfC Alloys Prepared by SLM

**Figure 7:**
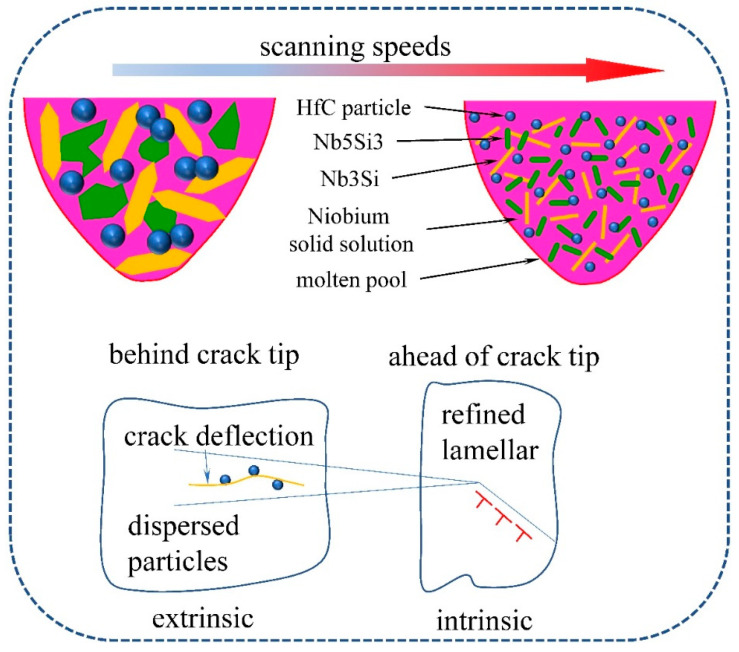
The schematic diagram of the toughening mechanism of Nb18Si5HfC alloy.

The fracture toughness at room temperature was computed at various scanning speeds, as shown in Table 1. As expected, scanning speed had a significant effect on the fracture toughness of the Nb-18Si-5HfC alloy and the maximum toughness of 20.7 MPa∙m^1/2^ was realized at a scanning speed of 1000 mm/s. Both intrinsic and extrinsic mechanisms [26,27] contributed to enhancing the indentation fracture toughness of the current hafnium carbide-distributed Nb-18Si alloy. The schematic diagram of the toughening mechanism of Nb-18Si-5HfC alloy is shown in Figure 7. Specifically, the intrinsic toughening mechanism is mainly reflected in the tuning of the scan speed to the dimensional morphology of the α-Nbss solid solution matrix. The α-Nbss matrix morphology gradually evolved into discontinuous nanoscale lamellae, and the aspect ratio of the lamellae was significantly reduced. This morphology not only blunted the crack tip, but also blocked the continuous crack expansion. Simultaneously, the hafnium carbide particles distributed in the eutectic interstices served as an effective external toughening mechanism. Uniform distribution of fine hafnium carbide particles not only played a role in deflecting cracks, but also improved toughness by introducing microcracks at eutectic interface to produce new surfaces, absorbing higher energy (indicated by the yellow arrow in Figure 5). Therefore, we believe that the fracture toughness of Nb-18Si-5HfC alloys has been improved by both intrinsic and extrinsic mechanisms through the design introduction of hafnium carbide particles and tuning of microstructure morphology.

## 5. Conclusions

The main findings in the work on the Nb-18Si-5HfC alloy, created by selective laser melting, are summarized in the following key points:

1. The dense Nb-18Si-5HfC alloys were successfully prepared by selective laser melting. The ultrafine microstructure modulation was achieved by adjusting the scanning speed from 600 mm/s to 1200 mm/s.
2. When the scan speed was higher than 1000 mm/s, the fine diffuse hafnium carbide particles successfully distributed between the eutectic lamella, which resulted in improved indentation fracture toughness.
3. The prepared Nb-18Si-5HfC alloy, at a scanning speed 1000 mm/s, had a maximum indentation fracture toughness of 20.7 MPa∙m^1/2^. The toughening was mainly attributable to the synergistic co-donation of the crack trapping by the refined discontinuous α-Nbss, and the crack deflection of the hafnium carbide particles by the formation of microcracks.

## Figures and Tables

**Table 1:** Fracture toughness calculated at different scanning speeds.

Processing Parameter (mm/s)	a (μm)	l (μm)	c (μm)	Hardness (GPa)	Elastic Modulus (GPa)	Fracture Toughness (MPa·m^0.5^)
600	20.9	10.2	41.2	4.82	123.6	7.4
800	20.9	12.1	32.9	6.48	142.9	8.6
1000	20.9	3.7	24.6	9.22	160.6	20.7
1200	20.9	4.5	25.4	13.83	230.5	17.3

## Data Availability

Some or all data, models, or code that support the findings of this study are available from the corresponding author upon reasonable request.
